# How has COVID-19 affected the work environment of delivery workers?: An interpretative phenomenological analysis

**DOI:** 10.1371/journal.pone.0290403

**Published:** 2023-09-21

**Authors:** Jeehee Pyo, Eun Jee Park, Minsu Ock, Won Lee, Hye Jin Lee, Sungkyoung Choi

**Affiliations:** 1 Task Force to Support Public Health and Medical Services of Ulsan Metropolitan City, Ulsan, Republic of Korea; 2 Department of Preventive Medicine, College of Medicine, Chung-Ang University, Seoul, Republic of Korea; 3 Department of Preventive Medicine, University of Ulsan College of Medicine, Ulsan, Republic of Korea; 4 Department of Nursing, Chung-Ang University, Seoul, Republic of Korea; 5 Department of addiction studies, The Catholic University of Korea, Bucheon, Republic of Korea; 6 Department of Nursing, Catholic Kwandong University, Gangneung, Republic of Korea; The University of Queensland, AUSTRALIA

## Abstract

The COVID-19 pandemic has sparked a rapid worldwide increase in the utilization of delivery services. This study delves into the experiences of delivery workers as one of the activley developed industries during the COVID-19 pandemic in South Korea and sheds light on the effects of the pandemic on their working conditions. Through in-depth interviews with 10 Korean delivery workers, data analysis employed the hermeneutic phenomenology research method developed by Van Manen. The findings indicate a substantial rise in income levels and a positive societal perception of delivery labor post-COVID-19. The pandemic also attracted many new workers to the industry due to low entry barriers and work flexibility. However, challenges persisted as delivery workers grappled with an uncertain legal status and sometimes jeopardized their safety to boost earnings in shorter time frames. The pivotal role played by delivery workers in enhancing communal quality of life and connectivity during the pandemic cannot be overlooked. As we step into a post-COVID-19 era, comprehensive efforts are needed to enhance the working environment for delivery workers globally. Notably, clarifying the relationship between delivery workers and companies within the novel digital labor landscape is essential, alongside establishing institutional frameworks to safeguard workers’ basic rights, including health and safety provisions.

## Introduction

During the COVID-19 pandemic, countries worldwide have implemented social distancing measures to protect their populations, leading to various changes for both individuals and communities [1]. These restrictions specifically led to a rapid increase in the consumption of goods and services online [2]. Consequently, the online-to-offline (O2O) service system, in which products purchased online are delivered to consumers’ homes, has grown significantly. South Korea, with its high internet penetration rate, is one of the countries with a vibrant online shopping culture. Its relatively small territory (in geographical terms) also enables it to enjoy a high-quality logistics infrastructure compared to other countries [3]. The combination of all these factors has contributed to explosive growth in the delivery industry in the wake of the COVID-19 pandemic. The online marketplace and, accordingly, the parcel delivery volume have increased by approximately 20% in 2020 compared to previous years [4], and the number of delivery workers also increased [5].

During the COVID-19 pandemic, delivery workers have played a central role in connecting everyone with the outside world by directly delivering daily necessities and groceries to people who have difficulty moving freely. Delivery workers are self-employed who use the platform to deliver food or goods, and have more flexibility and autonomy than traditional location-based workers [6,7]. Unfortunately, COVID-19 has made it difficult for delivery workers. They have been overworked due to a surge in deliveries, and their wages have lowered as a result of increased competition among delivery companies [8,9]. Additionally, delivery workers face a great risk of accidents and injuries because they spend most of their time on the road and are under pressure to deliver food and/or goods to consumers quickly. In fact, since 2019 in South Korea, motorcycle accidents have increased along with the rise in food delivery [10]. Most delivery workers are vulnerable workers who do not receive legal protection due to their unclear legal status, and COVID-19 has further exacerbated their vulnerability. In other words, while going through COVID-19, delivery workers were recognized as valuable occupations, but they also experienced a deterioration in their working conditions. The Post COVID-19 era will be a new era, different from before COVID-19. Uncertainties such as the rapid growth of online platforms, the spread of new forms of labor structures, and the risk of another infectious disease have made it more necessary to understand the various changes brought about by COVID-19.

However, previous studies on delivery work have mainly focused on labor structure, economy, consumer behavior or transportation [11–13]. After the COVID-19 pandemic, studies on the impact of COVID-19 on delivery workers have been published [14–17], but studies that directly explore their experiences and focuse on the human aspect of delivery workers are still limited. Therefore, this study aims to explore the experiences of Korean delivery workers and comprehend the influence of COVID-19 on their working environments from their perspective. This study findings add value to the literature to prepare for the post-COVID19 new normal era, and contribute to policy makers and experts who strive to improve the working environment of delivery workers as meaningful knowledge.

## Materials and methods

### Study design

Phenomenology, a traditional qualitative research method, aims at understanding the meaning of the human experience or the “life-world [18].” This research method examines the phenomenon (experience) as it occurs in the participant’s consciousness, excluding the researcher’s prior knowledge and/or prejudices, and describes the facts as they are [19]. In this study, the hermeneutic phenomenology method, developed by Van Manen, was selected from among the data analysis methods typically implemented in phenomenological research. It allows for the exploration of the kind of life-world the participants inhabit, their experiences in that life-world, what their life experiences have meant, as well as the essential meaning of these experiences [20]. Specifically, we performed the following steps in our research: (1) Selected a research topic based on our research interests. (2) Conducted an analysis of the essential elements related to the research topic that we wanted to investigate. (3) Within the components of the research topic, we selected core elements. (4) Developed semi-structured guidelines for explore the core elements. (5) Conducted semi-structued guidelines individual in-depth interviews(IDIs). The most widely used interview format in qualitative research, for data collection. IDIs allow the researcher to delve deeply into social and personal matters [21]. (6) Based on the data obtained from the IDIs, we derived and categorized meaningful units that align with the essential elements and described the results. Furthermore, the research team diligently implemented measures to ensure the validity, and reliability of the derived outcomes. We followed the Consolidated Criteria for Reporting Qualitative Research [21].

### Study participants

The criteria for participation in this study were: (1) adults aged 19 years and above, (2) delivery workers falling under classification code 922 of the Korean Standard Classification of Occupations (i.e. postmen, door-to-door deliverers, food deliverers, other deliverers), and (3) those who has been working as delivery workers since before the outbreak of COVID-19. The third criterion was set to compare the experience of delivery workers before and after the COVID-19 pandemic. Participants were recruited via advertisements posted on the online open messaging platforms of delivery companies accessible to delivery workers. Delivery workers who wished to participate voluntarily accessed the Google Form link provided in the advertisement and submitted their essential information. Subsequently, researchers called them, confirmed whether they met the participation criteria, explained the necessity and purpose of the study in detail, and confirmed the interview date. The researchers also asked participants to recommend colleagues for participant recruitment. The recruitment of participants was conducted simultaneously with the interviews until data saturation. A total of 20 delivery workers voluntarily applied for participation in the study, but 3 of them did not answer the phone call from the researchers, and 5 did not participate in the interview due to personal reasons. Two who belatedly applied for participation in the study after data saturation had already been reached participated in the review of the study results to certify the validation (Applicability) of this study.

### Data collection

Data collection occurred from December 19, 2020, to May 4, 2021, spanning a total of six months. Semi-structured IDIs were conducted by trained researchers (JP, EJP, and SC) who had experience in conducting qualitative studies. Each participant underwent at least one IDI session, with each session lasting approximately an hour. These IDIs were conducted online via Zoom meetings, in compliance with the COVID-19 guidelines at the time. Since the interviews were conducted online, the actual interview setting was entirely at the discretion of each participant. However, we recommended that all participants position themselves in a psychologically safe environment. Each interview began with an introduction by the researcher to establish rapport with the participant, creating a comfortable atmosphere through general conversation and inquiring about the participants’ current subjective health status. The researchers conducted interviews following the semi-structured interview guideline, which was developed through a comprehensive literature review. All interviews were recorded with the participants’ consent. Data collection and analysis were conducted almost simultaneously, and starting from the 6th interview, the semi-structured interview guideline was supplemented by reflecting the themes of the delivery workers’ experiences identified through data analysis (S1 Appendix). Each participant underwent one IDI session lasting approximately an hour, and for one participant (participant 4) who could not complete the statement he wanted to make in the first interview, the second interview was conducted for about 20 minutes the day after the first interview. After the interview, participants were rewarded with 100,000 KRW regardless of the number of interviews, as already explained to the participants in the consent process for the interview.

### Data analysis

The researchers analyzed the data by focusing on the phenomena the participants experienced with COVID-19. The recorded interviews were immediately transcribed, and the three researchers who conducted the interviews independently read the transcripts line by line and performed text separation work (bracketing and semantic unit derivation). Regular research team meetings were held to refine and interpret the semantic units extracted from the line-by-line analysis through an iterative and cyclical process [20]. After classifying the words and/or sentences that revealed the essence of the phenomena and experiences within the separated texts, theme statements were extracted from the classified words and sentences and converted into terms that accurately conveyed the meaning of these phenomenological experiences. The researchers also engaged in the hermeneutic phenomenological reflection stage during the subject analysis. Unclear contents and/or insufficiencies that were deemed relevant to the study theme were clarified through brief phone calls with the research participants.

### Validation of research

The validity of the research results was authenticated through the evaluation of the truth value, the applicability, consistency, and neutrality of the findings, utilizing a method developed by Guba and Lincoln [22]. To examine the truth value, we shared the qualitative study results with two participants (participant 1 & 5), and they reviewed whether their experiences and interviews were accurately reflected in the study results. To ensure the applicability, first, we conducted IDIs until data saturation, which occurs when no more new content emerged. Data saturation is ensured through the researcher’s experience, and it combines sampling, data collection, and data analysis as a continuous process rather than discrete steps [23]. Therefore, we conducted IDIs and data analysis almost simultaneously while continuously recruiting participants. Additionally, research team meetings were held periodically to assess whether newly collected data would contribute to new themes. Second, we ensured data saturation and applicability by asking two delivery workers (one delivery app worker and one courier) who met the participant selection criteria but did not participate in the study to review the study results. They provided feedback on the similarity between the study results and their own experiences. To ensure consistency, we presented the entire study process in detail in the paper, aiming to provide a thick description to the readers. Furthermore, we sought objectivity in the research process by continuously receiving peer-review from one qualitative research expert, one expert in the field of vulnerable workers, and one preventive medicine specialist. Neutrality was managed through the sharing of the researchers’ initial theme-related understandings and assumptions prior to the commencement of the study (Table 1). Before starting the IDIs, we held a total of 12 meetings from April 2020 to November 2020 to review and discuss the qualitative research methodology and ethical issues. Additionally, to eliminate researcher prejudices, an epoché (the suspension of judgment) was conducted by reviewing their prior understandings, biases, and assumptions, and “bracketing” was performed during the data analysis stage.

**Table 1:** The researcher’s prejudice related to the topic.

1. Delivery workers will be experiencing precarious employment and social security deficiencies in the Covid-19 situation. 2. Economically, positive changes are expected due to an increased workload stemming from COVID-19. 3. In terms of health, the workload will increase due to COVID-19, potentially leading to a lower level of subjective health-related quality of life. 4. Due to the Covid-19 situation, their general lives such as health and economy must have changed greatly.

### Ethics approval

This study was conducted taking into consideration the requests, the physical, and psychological health of all participants. Prior to consenting, the purpose of the study, method of participation, and reimbursement were discussed in detail with the participants verbally and in writing. All participants provided written informed consent prior to enrollment in the study. The contents of the IDIs were used for research purposes only. The participants were also free to choose whether to participate or withdraw from the study at any time (though they would not be compensated prior to completing the IDIs). As different participants were affiliated with different companies, we reassured the participants that the data would be kept in the strictest confidentiality and that pseudonyms would be used to protect their anonymity. This study was approved by The Institutional Review Board at Ulsan University Hospital (IRB No. 2020-11-016).

## Results

Table 2 presents the information of the 10 participants who ultimately took part in the study. There were 5 couriers, 4 delivery app workers, and 1 restaurant employee, and their average age was 35.7 years old. The average age of couriers was approximately 10 years older than that of delivery app workers. Couriers mainly utilized trucks for parcel deliver, while delivery app workers mainly utilized motorcycles for food delivery. Their delivery experience ranged from 1 year and 5 months to 17 years.

**Table 2:** Characteristics of study participant.

No	Sex	Age (years)	Regions	Duration of Work	Status^a^	Delivery item	Delivery vehicle
1	Male	33	Incheon	1 year 5 months	Delivery app worker	Food	Motorcycle
2	Female	43	Gyeonggi	4 years	Courier (logistics service company)	Parcel	Truck
3	Male	28	Daejeon	2 years	Chinese restaurant employee	Food	Motorcycle
4	Male	33	Daegu	2 year 2 months	Delivery app worker	Food	Motorcycle
5	Male	41	Chungnam	15 years	Courier (logistics service company)	Parcel	Truck
6	Male	47	Pusan	4 years	Courier (logistics service company)	Parcel	Truck
7	Male	25	Seoul	6 years	Delivery app worker	Food	Motorcycle
8	Male	36	Daegu	3 year 6 months	Courier (post office)	Parcel	Truck
9	Male	37	Gyeonggi	4 years	Courier (post office)	Parcel	Truck
10	Male	34	Seoul	17 years	Delivery app worker	Food, Document	Motorcycle

^a^ Delivery app worker refers to a self-employed individual who works as a delivery driver for a delivery app platform.; Courier refers to a self-employed individual who contracts with a logistics service company or post office to undertake parcel transportation.

We identified a total of 139 semantic units, which were grouped into eight sub-themes and three themes (Table 3).

**Table 3:** Qualitative themes and subthemes.

Theme	Sub-theme
1. Increased social value of delivery after the COVID-19 outbreak	1–1. An increase in the use of delivery services to prevent COVID-19 exposure 1–2. Increase in the number of people wanting to work in the delivery industry
2. A more enjoyable place to work in	2–1. Customers actively expressed appreciation 2–2. Easier and more accessible work due to non-face-to-face delivery 2–3. Revenue increases due to daily delivery
3. More motivation to work but safety is more at risk	3–1. Experiencing physical and psychological difficulties due to increased workload 3–2. Concerns about COVID-19 infection 3–3. Increased risk of road accidents due to voluntary and/or involuntary pressures

### Theme 1: Increased social value of delivery after the COVID-19 outbreak

#### Subtheme 1–1: An increase in the use of delivery services to prevent COVID-19 exposure

Participants commonly reported that the volume of delivery increased after the COVID-19 outbreak. They said “Prior to the COVID-19 outbreak, approximately three days a week were physically less challenging days”; however, after the outbreak, they became busy “every day of the week.” Delivery zones also expanded to include rural and outer areas not serviced prior to the outbreak. This is because the increase in orders for single-person households and social distancing led to the expansion of delivery volume and delivery areas. This made it easier for delivery workers to plan their routes. Furthermore, Courier participants mentioned that they experienced a sense of novelty due to the diversification of items being delivered. In the past, they primarily delivered items that were difficult to purchase within their local area. However, after the outbreak, they mentioned that they began delivering items such as cosmetics and groceries that are easily available within their local area.

*“Under Social Distancing Level 2*.*5*, *restaurants are allowed to serve food after nine in the evening only via delivery*. *There are a lot of deliveries*, *and the number of orders is increasing as it gets colder*. *Yesterday*, *there were backlogs of about 150 orders*.*”* [Participant 1]

*“I’ve heard that some people have had to quit their jobs because of COVID-19*, *but in my case as a delivery worker during the pandemic*, *I actually think it’s a prosperous time for my career*..*”* [Participant 3]

#### Subtheme 1–2: Increase in the number of people wanting to work in the delivery industry

While many people struggled economically due to COVID-19, the delivery industry was booming. As a result, many people have entered delivery labor, which has relatively low entry barriers and guarantees work flexibility. Participants reported receiving inquiries regarding employment availabilities from friends and families and that close acquaintances had also “jumped into the delivery business.”

*“I think*, *as it has become more hard to make ends meet these days*, *more people are engaging in delivery work than before*. *For example*, *your friend may be working as a delivery worker or a friend of your husband may be working as a delivery worker*. *It could be anyone in a social circle*.*”* [Participant 4]

### Theme 2: A more enjoyable place to work

#### Subtheme 2–1: Customers actively expressed appreciation

Participants mentioned that in the past, they couldn’t feel proud of themselves due to the negative perception people had towards delivery workers, such as being seen as aggressive drivers. However, post-COVID-19, participants observed a significant shift. They mentioned experiencing expressions of gratitude from customers on a frequent basis. Customers were also more compassionate as evidenced by some customers saying “since it’s raining, you could deliver slowly—I don’t mind the food getting a little cold,” and “thank you for your service in such cold weather.” Receiving tips or snacks also became more common and some participants reported that this kindness enabled them to enjoy their work.

*“Things have gotten much better for us*. *People don’t seem to have a negative perception of delivery work…More people seem to think that we work hard and help people*.*…During my working hours*, *there are constantly people handing me beverages*, *tangerines*, *or bananas*. *Yesterday*, *I received a banana*, *and the customer said*, *‘thank you for your service in such cold weather’*.*”* [Participant 1]

*“The customer thanked me for the delivery and handed me a drink*.*”* [Participant 5]

However, some participants experienced being treated as potential spreaders of infection by citizens or customers they encountered during the delivery process.

*“Well*… *there are customers who appreciate our services*, *but there are also people who regard us as filthy*. *You know*, *there were people who said this*, *‘Don’t get in the same elevator as me*. *Take the other elevator after I go first’*.*”* [Participant 8]

#### Subtheme 2–2: Easier and more accessible work due to non-face-to-face delivery

After COVID-19, conventional face-to-face delivery services have been replaced by contactless deliveries using unmanned delivery boxes or leaving goods and/or food items at the front door or security office for the safety of both delivery workers and customers. The participants believed that these changes had an impact on the decrease in customer’s complaints, although they couldn’t pinpoint the specific reasons. Participants mentioned that contactless delivery increases profitability by shortening delivery times, enabling more deliveries to be processed in the same amount of time. They also said that their working environment has improved because they no longer have to interact with customers in person, resulting in less stress.

*“Face-to-face deliveries take a lot of time waiting for the customers come out*. *Non-face-to-face deliveries are much easier*. *I can leave the delivery in front of the house*. *That’s all*. *As I told you earlier*, *every second counts for us*. *Every second*. *This saves a lot of time*. *To be honest*, *it is very convenient for us*.*”* [Participant 9]

#### Subtheme 2–3: Revenue increases due to daily delivery

Couriers, who receive parcels from logistics companies and deliver them to customers, reported that their income had increased by approximately 1 to 2 million KRW during COVID-19 outbreak. Delivery app workers, who mainly deliver food, also reported an income increase of around 30%. Some delivery app workers mentioned that COVID-19 has brought us significant income potential, which motivated me to become involved in delivery work. Participants described the COVID-19 era as a “good environment for the delivery business” and a “period of prosperity.”

*“There was about a KRW 1 to 2 million increase (in income) and our income is expected to increase even more during the peak season*.*”* [Participant 7]

*“This is a good time to earn money…Under Social Distance Level 2*.*5*, *restaurants can serve food after nine in the evening only via deliveries*. *There is a high demand for delivery services*, *and this demand has become even higher as it is getting colder outside…We seem to be in a rather good environment (to make money) right now*.*”* [Participant 1]

### Theme 3: More motivation to work but increased safety risks

#### Subtheme 3–1: Experiencing physical and psychological difficulties due to increased workload

There was a general consensus among participants that their physical and mental health had suffered since the COVID-19 outbreak. They didn’t always get enough rest or meals, and they were unable to finish work on time due to the large number of deliveries to be completed. The increase in deliveries to multi-level buildings without elevators worsened participants’ physical health.

They also raised the issue of the increasing difficulty of finding time to spend with family and friends. Several delivery app workers expressed feeling mental pressure stemming from the system in which the company that operates the delivery app platform forcibly assigns excessive deliveries to them. In summary, the participants experienced increased stress due to higher delivery volumes and time pressure. As a result, some participants smoked more cigarettes and engaged in more frequent traffic violations. Throughout this process, they perceived a decline in their physical and mental well-being.

*“When I look back on my life*, *I’ve been very busy*. *It has been very difficult*. *As for my personal relationships*, *I’m running out of time and it’s almost 9 or 10 when I get home*, *which makes it hard for me to spend time with my baby and my wife*. *Life has become a little harder in that way*.*”* [Participant 2]

#### Subtheme 3–2: Concerns about COVID-19 infection

Participants were concerned about the risk of becoming infected with COVID-19, which was heightened by the increased work volumes and delivery demands. Some participants reported constant anxiety about having to work in COVID-19 outbreak hotspots. They were unable to use the masks provided by the company as the sizes didn’t fit them properly. Additionally, some participants had to deliver parcel or food to homes where people with COVID-19 were in quarantine. They felt fear because they didn’t receive any relevant information.

*“I try to avoid face-to-face deliveries*, *and I use alcohol*, *you know*…*hand sanitizer*. *I used it all the time*.*”* [Participant 4]

“*There were cases in which those under quarantine for COVID-19 opened the door to receive the delivery items*.*”* [Participant 6]

#### Subtheme 3–3: Increased risk of road accidents due to voluntary and/or involuntary pressure

Following the increase in delivery volumes after the COVID-19 outbreak, some participants admitted to violating several traffic signals to ensure a quick delivery. As previously mentioned, this not only contributed to increased psychological stress but also increased the risk of actual traffic accidents. Specifically, during peak hours of food delivery, there were participants who had to deliver up to 6 meals at a time due to the forced immediate dispatch system. Complaints from customers demanding fast delivery also made them violate traffic signals.

On the other hand, there were many cases where participants voluntarily violated traffic signals to increase their income, despite no external pressure. Some participants mentioned that they had actually experienced road accidents while working as delivery workers, and there were also cases where their colleagues had died as a result of road accidents. Therefore, they were aware of the risks associated with road accidents.

*“In a rush*. *Everyone is driven by greed for money*. *It’s just that due to the desire of earning money*, *accidents happen when they try to make a lot of deliveries quickly*.*”* [Participant 1]

*“It’s not that I intentionally wanted to deliver late*, *but it’s frustrating when customers get angry just because I was a few minutes late*. *And because of the delayed delivery*, *some people even give low ratings to the restaurant on the delivery app platform*.*”* [Participant 3]

## Discussion

This study was aimed to explore the experiences of delivery workers, who played an extremely important role during the COVID-19. One of our findings was that the pandemic had not been entirely negative from the perspective of certain job market industries. Since the COVID-19 outbreak in December 2019, the world has experienced a global economic crisis, which had a significant negative impact on the general labor market. However, with the O2O service system becoming inevitable during the COVID-19 era, the delivery labor field experienced a boom with the increase in delivery volume. Delivery labor does not require professional skills or education, and anyone with a driver’s license can do it [24]. Therefore, compared to other jobs, delivery workers were relatively young, had a low level of education, and were recognized as having poor working conditions [25,26]. However, after COVID-19, the income level of Korean delivery workers has risen considerably, and there has been a positive perception of delivery labor throughout society. According to a survey conducted by a research institute in South Korea [27], most people were grateful for the delivery service that was operated without any setbacks in the midst of the COVID-19 outbreak. Many customers supported raising delivery costs and even proposed the introduction of a public holiday exclusively for delivery drivers. This can be seen as a re-evaluation of the significance of the labor performed by delivery workers, along with a heightened societal awareness in the face of an unprecedented situation. Additionally, contactless delivery, introduced to prevent COVID-19 infection, seems to be a factor that has increased the positive perception of delivery workers. As individualized lifestyles are strengthened and interaction avoidance is preferred due to COVID-19 [28], non-face-to-face relationships provide customers with more psychological stability than direct interactions [29]. Contactless delivery has also contributed to improving the income and work environment of delivery workers by reducing unnecessary contact with customers and enabling more deliveries in a limited time. However, previous studies in China, Brazil, and India that explored the working conditions of delivery workers showed contradictory results to this study. They report that the profitability of delivery workers has declined during COVID-19 due to the cost of efforts to protect against infection and competition among many delivery workers [14,30–32]. To the best of our knowledge, this study is the first to explore the experiences of delivery workers in South Korea. The social impact of COVID-19 and the delivery labor structure may differ from country to country. Therefore, future comparative studies by country may be helpful in finding ways to improve the working environment of delivery workers.

With the digital transformation, an economic structure has emerged in which companies utilize online platforms to promote and offer self-employment opportunities to individuals, providing them with temporary, flexible, or freelance jobs (termed the “gig economy”), which is more popular than ever [33,34]. In this labor structure, workers have the freedom to provide labor on their own terms and have more flexibility and autonomy compared to traditional location-based workers [6,7]. Workers who deliver goods to consumers as part of the O2O system are an important group that supports the overall gig economy. Due to COVID-19, many people started working as delivery workers, and this change is not unique to South Korea [31]. We note that a high proportion of them are young people. One of the defining features of the gig economy is its flexibility. Workers take charge of their own schedules and have the ability to work on their own terms. The influx of young people into delivery workers may reflect the preferences of a new generation who have gradually abandoned the traditional concept of having “a job for life” and prioritize their identity over being bound by external restrictions or rules [35]. Conversely, it can also be viewed as a phenomenon in which the less-skilled, younger generation, experiencing difficulties in entering an economically downturned labor market caused by the pandemic, join the delivery industry where there is high-income earning potential despite low entry requirements [36]. These young delivery workers often perceive the delivery business as a transitional job [30]. Delivery workers have become indispensable throughout the pandemic, greatly improving communal quality of life and connectivity. As online-oriented consumption behavior expands further with the development of digital technology [37], the importance of delivery worker will continue even after the COVID-19 ends. Therefore, it is necessary to explore the causes of delivery workers’ turnover and prepare improvement measures for them. According to Nguyen’s research, job burnout is the biggest factor that causes them to quit their jobs [26], and there are other studies on the poor working environment of delivery workers, such as racial discrimination [14,38].

In particular, unstable working conditions of delivery workers are commonly pointed out in studies conducted in various countries [39–42]. It is known that the gig economy provides flexibility and autonomy, but as our research results show, delivery workers often face work-related pressure from companies with which they signed contracts, as if they were employees. However, since the legal status of delivery workers is as independent contractors, the company has no obligation to guarantee worker benefits such as insurance, paid vacation, and protective equipment. Previous studies have described delivery workers in ambiguous situations as ‘unbelonging [40]’ or ‘disguised employees of the companies [41],’ and have warned against overemphasizing the advantages of the gig economy [43]. Nevertheless, fortunately, the participants in this study were provided with basic protective equipment such as masks from companies during the COVID-19 period. In a study conducted on bicycle delivery workers in Canada, 71.4% of delivery workers received masks or gloves to prevent COVID-19 infection [17]. However, it is still rare for most countries to provide them with protective equipment or hygiene products [44]. Delivery workers can become ’super spreaders’ because they travel to many areas and come into contact with many people. When companies value and support worker rights, delivery workers comply with COVID-19 regulations, and their health problems are reduced [15,24]. Therefore, it is necessary to clarify the relationship between delivery workers and the company, which is a new labor structure in the digital age, and to establish an institutional arrangement to guarantee basic rights to workers, such as health and safety.

The instability of delivery workers is not simply caused by an ambiguous legal status. Delivery work is often accompanied by strong time pressure [14], which puts delivery workers in dangerous situations on the road. Violation of traffic signals by delivery workers occurs involuntarily due to pressure from customers or excessive workload [32], but sometimes it occurs voluntarily. Being able to earn an income proportional to their effort is the biggest advantage of delivery labor. However, it is also a key factor that threatens the safety of delivery workers. They may choose to risk their own safety to make more income in a shorter period of time. One of the study participants (Participant 1) reported that a colleague died while violating a traffic signal in order to earn more money. Transportation (e.g., motorcycles and trucks) is characteristic of delivery labor, and thus there is always the risk of traffic accidents. Therefore, it is necessary to establish a delivery culture that values safety over delivery speed. Most of the previous studies on road safety of delivery workers dealt with the current status of signal violations [45,46]. Studies on the relationship between delivery workers’ desire for income and their road safety are needed. Meanwhile, this is also associated with the professional ethics of delivery workers. Risk and safety perceptions, in terms of road safety and rules, can be cultivated through education [47]; therefore, efforts such as developing a training course for these workers can be extremely helpful in promoting worker and overall public health.

This study has several limitations. First, we interviewed workers within various occupational groups within the delivery industry (courier of logistics service company or post office, delivery app worker, and restaurant employee). Therefore, there was a limit to an in-depth investigation of the characteristics of each occupational group. Second, the gender of the enrolled study participants was biased towards men. Although there could well be a difference in the experiences between men and women employed in the same or similar type of work, only one woman participated in this study. However, we note that according to data released by Statistics Korea in 2020 [5], the male to female ratio of delivery laborers is 9:1. Thus, the comparative proportion of men and women among the participants enrolled in this study is consistent with that ratio and is then likely reflective of the actual distribution of workers in this profession. Nevertheless, this qualitative study has informative value for other countries because it was conducted in South Korea, a country with an advanced delivery culture, as well as a systematically and exponentially developing logistics and distribution system. This is a meaningful study that deeply explored the experiences of delivery workers in the COVID-19 pandemic situation from a personal, human perspective.

## Conclusion

Since the COVID-19 crisis, delivery workers in South Korea have experienced positive changes such as increased income and improved social awareness. However, along with the active and important role of delivery, numerous problems arising from the unstable legal status of delivery workers have also come to the surface. COVID-19 has brought significant changes to our daily lives, and the post COVID-19 era will be a new normal. With the expansion of the online market and the O2O service system becoming a part of our daily life, the importance of delivery drivers will continue even after the pandemic ends. To prepare for the future, multi-faceted efforts will be needed to improve the working environment of delivery workers.

## Supporting information

S1 Appendix Semi-structured interview guideline. (DOCX)
