# Supplementary material for: How has COVID-19 affected the work environment of delivery workers?: An interpretative phenomenological analysis
Source: PLoS One. 2023 Sep 21;18(9):e0290403. doi: 10.1371/journal.pone.0290403 (PMC10513220; doi:10.1371/journal.pone.0290403)
Supplement: S1 Appendix — (DOCX) [file pone.0290403.s001.docx]

**S1 Appendix. Semi-structured interview guideline.**

January 02, 2021

| Main questions and additional questions | |
| --- | --- |
| Part 1 | 1. What was your awareness of the occupation of a delivery worker? (before and after becoming a delivery worker)  2. How has delivery work affected your health? (physical, mental and social health)  3. What do you think about the working conditions of delivery workers?  4. Could you negotiate working conditions with the contracting company? |
| Part 2 | 5. How did you become a delivery worker?  6. Do you have any memorable experiences working as a delivery worker? |
| Part 3 | 7. Do you have any memorable experiences working as a delivery worker after the outbreak of COVID-19?  8. Has there been any change in delivery work since the outbreak of COVID-19? what kind of change?  9. Has your awareness of the occupation of a delivery worker changed since the outbreak of COVID-19? |
| Part 4 | 10. What does delivery work mean to you?  11. Please tell us about what needs to be improved in the field of delivery work.  12. Please tell us about what needs to be improved in the field of delivery work, especially in a pandemic like COVID-19. |
